# Elevated regulatory T cells, surface and intracellular CTLA-4 expression and interleukin-17 in the lung cancer microenvironment in humans

**DOI:** 10.1007/s00262-016-1930-6

**Published:** 2016-11-19

**Authors:** Iwona Kwiecien, Anna Stelmaszczyk-Emmel, Malgorzata Polubiec-Kownacka, Dariusz Dziedzic, Joanna Domagala-Kulawik

**Affiliations:** 1 Department of Pathomorphology, PhD Study (First Faculty of Medicine), Medical University of Warsaw, 7 Pawinskiego Street, 02-106 Warsaw, Poland; 2 Department of Laboratory Diagnostics and Clinical Immunology of Developmental Age, Medical University of Warsaw, 63a Zwirki i Wigury Street, 02-091 Warsaw, Poland; 3 Department of Surgery, Institute of Tuberculosis and Lung Diseases, 26 Plocka Street, 01-138 Warsaw, Poland; 4 Department of Internal Diseases, Pneumonology and Allergology, Medical University of Warsaw, 1a Banacha Street, 02-097 Warsaw, Poland

**Keywords:** T regulatory cells, CTLA-4, IL-17, Bronchoalveolar lavage, Lung cancer

## Abstract

Regulatory T cells (Tregs) play an important role in the suppression of the immune response in lung cancer. Cytotoxic T-lymphocyte antigen 4 (CTLA-4) expressed on T lymphocytes is capable of downregulating cytotoxic T cells and is constitutively expressed on Tregs. Little is known about the population of Tregs with two forms of CTLA-4: surface (s) and intracellular (in) in the lung cancer environment. Th17 cells defined by production of IL-17 have pleiotropic functions in anticancer immune response. Our aim was to detect the elements of immune response regulation in lung cancer in three compartments: by analysis of bronchoalveolar lavage fluid (BALF) from the lung affected by cancer (clBALF), healthy symmetrical lung (hlBALF) and peripheral blood (PB) from the same patient. A total of 54 samples were collected. Tregs, (s)CTLA-4, (in)CTLA-4 were detected by flow cytometry with antibodies against CD4, CD25, Foxp3, CD127, CTLA-4, and concentration of IL-17 was estimated by ELISA. We observed a significantly higher proportion of Tregs in clBALF than in hlBALF or PB (8.5 vs. 5.0 vs. 5.1%, respectively, *p* < 0.05). The median proportion of (in)CTLA-4+ Tregs was higher in clBALF than in hlBALF or PB (89.0, 81.5, 56.0%, *p* < 0.05). IL-17 concentration was the highest in clBALF—6.6 pg/ml. We observed a significant correlation between the proportion of Tregs and (in)CTLA-4+ Tregs with IL-17A concentration in clBALF. We confirmed significant differences in the proportion of regulatory elements between cancerous lung and healthy lung and PB and the usefulness of BALF analysis in evaluation of immune response regulation in local lung cancer environment.

## Introduction

Lung cancer is still the main oncological problem worldwide. There are about 1.8 million new cases yearly [1]. The prognosis is very poor with <15% of the overall survival. This cancer is the first cause of cancer deaths among patients with malignancy. About 70% of cases are in the advanced stages of this disease at the time of diagnosis, and these tumors are unresectable [2, 3]. Rapid accurate diagnosis, recognition of risk factors and the improvement of treatment efficacy are the main challenges in the case of this tumor. Many inhibitory mechanisms of immune response have been demonstrated in lung cancer patients in circulation and the resected tumor samples being the basis of new methods of immunotherapy [4–6]. The investigations of the possible biomarkers before immunomodulatory treatment are ongoing.

The impairment immune surveillance plays an important role in the progression of lung cancer. It is well known that T regulatory cells (Tregs) play a crucial role in inhibition of the immune response [7]. Their function depends on the expression of transcription factor Forkhead box P3 (Foxp3) [8]. Recently, the presence of Cytotoxic T-lymphocyte antigen-4 (CTLA-4) molecule has been demonstrated as a strong inductor of Tregs function [9, 10]. Tregs form a subpopulation of T cells, which modulate the immune system, maintain tolerance to self-antigens and abrogate autoimmune diseases. Tregs represent about 5% of circulating CD4+ T lymphocytes in the human peripheral blood and are defined by the expression of CD4, high expression of CD25 and Foxp3 and lack of CD127 [11]. Generally, Tregs suppress or downregulate the proliferation and function of effector T cells; thus, they may stimulate cancer progression [12]. An increased number of Tregs has been observed in the blood and in the tumor mass of patients with different solid tumors [13–15].

CTLA-4 also known as CD152 is a protein receptor that downregulates the immune system. CTLA-4 is constitutively expressed on Tregs. However, CTLA-4 can also function in the non-Treg compartment being expressed on other T cells [16]. CTLA-4 is similar to the T cell co-stimulatory protein, CD28. CTLA-4 and CD28 bind to CD80 or CD86 on antigen-presenting cells. CTLA-4 transmits an inhibitory signal, whereas CD28 transmits an activation signal to T cell [17]. CTLA-4 is identified as a surface or intracellular antigen. The physiologic regulation causes the immediate endocytosis of surface molecule, and the majority of CTLA-4 is localized in intracellular granules [18]. Thus, there are two forms of CTLA-4 expression: on the cell surface after activation (s), and intracellular as storage (in) [19].

The increased proportion of Tregs, high expression of Foxp3 and CTLA-4 on tumor infiltrating and peripheral blood lymphocytes have been observed in lung cancer [8, 10, 20]. Yet, little is known about the lung population of Tregs with CTLA-4 expression in the lung cancer microenvironment and the difference between (s) and (in) CTLA-4 on Tregs, which may be important for their function.

Th17 cells are defined by production of IL-17 and are known to be very plastic cells [21]. Some data show that IL-17 may play a dual role in the antitumor immunity [22]. IL-17 promotes an antitumor cytotoxic T cell response leading to tumor regression. On the other hand, by facilitating angiogenesis and egress of tumor cells from the primary focus, IL-17 promotes tumor spread [23–25].

The recognition of mechanisms of anticancer immune response may have important therapeutic implications [26]. Knowledge of the defense mechanisms in lung cancer is restricted to the studies of peripheral blood (PB), which reflects a systemic immune response and to a small number of non-small cell lung cancers (NSCLC), which are qualified to resection. Bronchoalveolar lavage (BAL) is a method for investigation of large part of the lung and enables examination of the local immune response in the lung cancer environment. BAL may be performed in all lung cancer stages [27]. All these reasons justified the choice of the BAL fluid (BALF) analysis as a basic material of this research.

The aim of the study was to detect some selected elements of the regulation of immune response in lung cancer based on the examination of BALF from the lung affected by cancer (clBALF as the local environment), from the healthy lung (hlBALF as a control) and in PB (as the systemic environment) from the same patient. We investigated the proportion of Tregs, Tregs with (s) and (in) CTLA-4 expression and IL-17 concentration in these three compartments, and we evaluated the relations between them.

## Patients and methods

### Patients

The study group consisted of 18 patients with confirmed primary lung cancer; in all patients it was NSCLC. There were 6 women and 12 men; mean age 68.4 ± 7.3 years; range (min–max) 50–81 years. There were patients in the I–III stages of the disease (according to 7th TNM classification) [28].

All patients underwent clinical examination, bronchoscopy with BALF (Department of Surgery, National Institute of Tuberculosis and Lung Diseases, Warsaw, Poland) and laboratory tests. Patient’s informed consent (the Medical University of Warsaw Ethics Committee) was required from all patients before each diagnostic procedure. Primary lung cancer confirmed by histological examination constituted an inclusion criterion. Exclusion criteria were as follows: any kind of anticancer therapy, signs of infection, chronic obstructive pulmonary disease (COPD), autoimmune diseases, immunosuppressive therapy. Further exclusion criteria were established after macro- and microscopic BALF examinations and those were: bloody fluid, <50% of recovered fluid, the absence of lymphocytes in the cytological samples in the May-Grunwald Giemsa (MGG) staining and no representative number of events in flow cytometric analysis of material.

### Bronchoalveolar lavage fluid

Bronchoalveolar lavage was performed in all patients during a routine diagnostic bronchofiberoscopy. During the procedure, 100 ml of 0.9% NaCl solution was instilled to each lung. BALF was taken from the cancerous lung (clBALF) and from the healthy lung (hlBALF) of the same patient during the same procedure. The volume of recovery fluid was 50% or more. BALF processing was realized according to the recommendations [29]. The material was filtered through a nylon gauze, the volume was measured, and then the fluid was centrifuged for 10 min (300×*g*). The cell pellet was used for total and differential cell counting and for flow cytometry. BALF supernatants were frozen at −70 °C and preserved until further processing. The Bürker chamber was used to measure the total cell count. Differential cell count was determined on two MGG-stained slides with the use of light microscopy. The rest of the pellets were used for cell analysis by flow cytometry.

### Flow cytometry analysis

Flow cytometry was used to determine the lymphocyte subtypes in the clBALF, hlBALF and PB. The numbers of Treg subpopulations and Tregs with the presence of CTLA-4 in samples were determined by a panel of monoclonal antibodies against: CD4 PE-Cy7, CD25 PE, CD127 BV421, Foxp3 Alexa Fluor 488, CTLA-4 APC (BD, USA); Tregs were defined as CD4+ CD25^high^Foxp3+ CD127-cells. The amounts of Tregs were presented as a median proportion of CD4-positive cells. Two forms of CTLA-4: surface (s) and intracellular (in) were analyzed in different tubes. For Foxp3 and (in)CTLA-4 detection, membrane permeabilization with Transcription Buffer Set (BD, USA) was used. CTLA-4-positive cells were shown as a median proportion of Tregs. The samples were processed by the FACS Canto II flow cytometer (BD, USA).

### Enzyme-linked immunosorbent assay (ELISA)

The BALF and serum IL-17A concentration was measured by means of a commercially available kit Quantikine ELISA Human IL-17A Immunoassay (R&D System, USA) according to the producer’s instruction. The absorbance was measured at 450 nm by a Microplate reader (the StatFox-2100 model; Awarness Technology Inc).

### Statistical analysis

The Statistica 12.0 software (StatSoft) was used for a statistical analysis. For group comparison, the Mann–Whitney test and Kruskal–Wallis test were used. Relations between quantitative variables were analyzed by Spearman correlations. A *p* < 0.05 was considered as statistically significant.

## Results

The characteristics of the investigated group are summarized in Table 1. In the investigated group, the men were significantly more numerous compared to the women and the male patients were significantly younger than female (*p* < 0.05). The prevalence of squamous cell carcinoma (50.0%) was noted. There were patients in stage I–IIIA of the disease (I-22.2%, II-61.1% and IIIa-16.7%, respectively). Due to a small number of patients in each group, we did not perform a comparison between the groups with different histological types of cancer and between different stages of the disease. There were 6 active smokers, 8 ex-smokers and only 4 patients who had never smoked cigarettes.

**Table 1:** Characteristics of the study population

	Patients
Sex F/M (*n*)	6/12
Age (mean ± SD years)	68.4 ± 7.3
Women (mean ± SD years)	72.3 ± 7.3
Men (mean ± SD years)	66.4 ± 6.7
Smokers/ex-smokers/non-smokers (*n*, %)	6 (33.3%)/8 (44.4%)/4 (22.2%)
Pack/years (mean ± SD)	36.3 ± 14.5
Histology (%)	
Squamous cell carcinoma	50.0%
Adenocarcinoma	22.2%
NOS	22.2%
Stage of disease (*n*, %)	
I	4 (22.2%)
II	11 (61.1%)
IIIA	3 (16.7%)
Symptoms of the respiratory system (interview of the patient) [yes/no (*n*, %)]:	4 (22.2%)/14 (77.8%)
Cough	2 (11.1%)/16 (88.9%)
Hemoptysis	1 (5.6%)/17 (94.4%)
Dyspnea	2 (11.1%)/16 (77.8%)

BALF description	clBALF	hlBALF
Total cell count (×10^6^)	7.1 ± 3.4	5.2 ± 2.7
MGG staining (mean ± SD)		
Macrophages (%)	49.5 ± 24.6	54.1 ± 23.2
Lymphocytes (%)	28.4 ± 14.3	22.2 ± 10.2
Neutrophils (%)	22.5 ± 21.7	23.6 ± 22.5
Eosinophils (%)	0	0

*BALF* bronchoalveolar lavage fluid, *clBALF* bronchoalveolar lavage fluid from the lung affected by cancer, *hlBALF* bronchoalveolar lavage fluid from healthy lung, *F* female, *M* male, *MGG* May-Grunwald Giemsa staining, *NOS* not otherwise specified

In the BALF analysis, the total cell count and differential cell count were similar when the clBALF and hlBALF were compared. There was an elevated proportion of neutrophils in the BALF: in the clBALF—22.5 ± 21.7%, in the hlBALF-23.6 ± 22.5%. The reference value of the proportion of BALF neutrophils is <3%) [29].

**Fig. 1:**
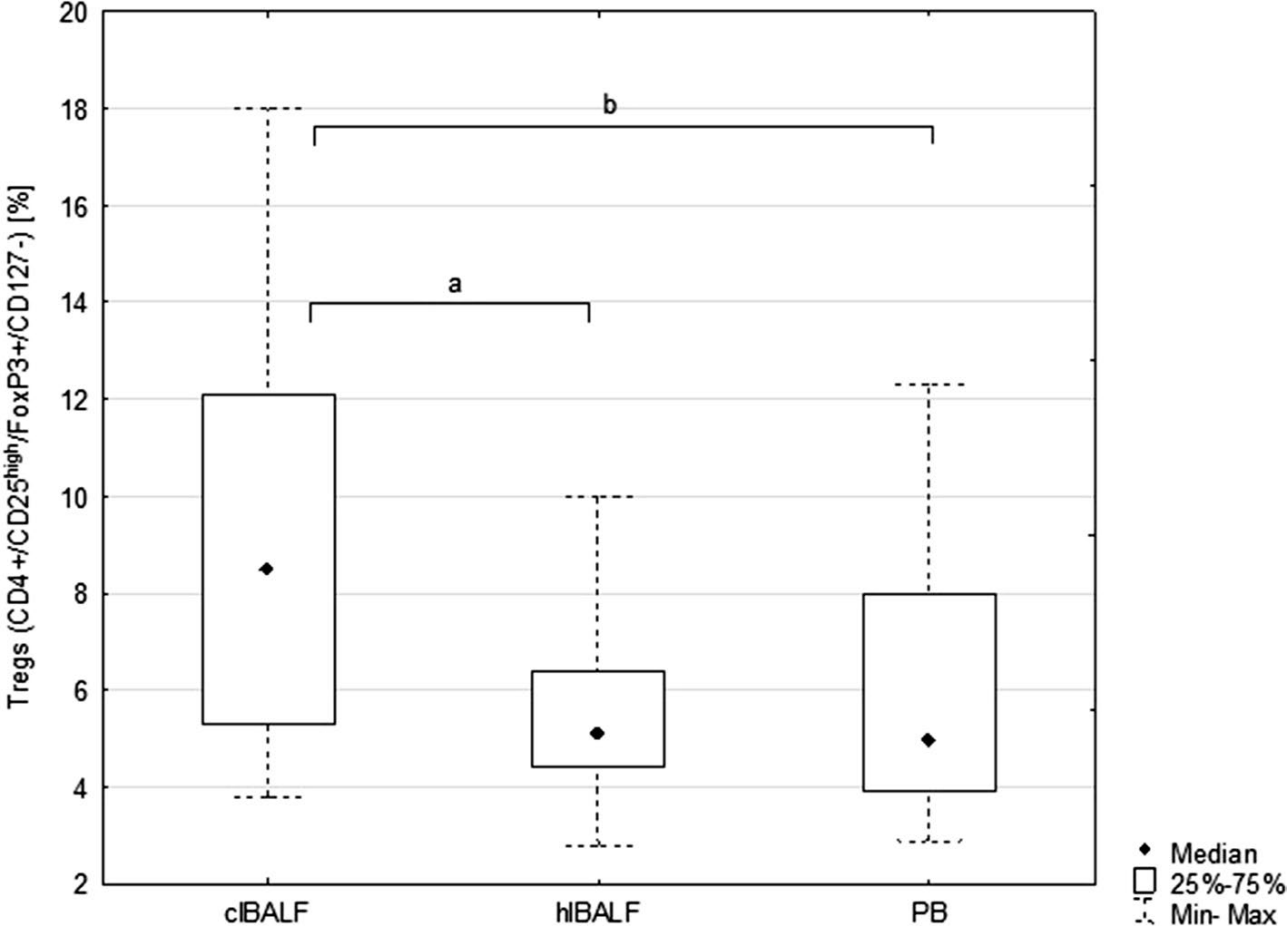
Median proportion of T regulatory cells (Tregs) in bronchoalveolar lavage fluid (BALF) of lung cancer patients from the lung affected by cancer (clBALF), from healthy symmetrical lung (hlBALF) and in peripheral blood (PB). (*a*, *b*: *p* < 0.05). Data are presented as median proportion, range p 25–75 and min–max

The proportion of Tregs in the clBALF was 8.5% (p25–p75 = 5.3–12.1) and was higher than in the hlBALF—5.0% (3.9–8.0) *p* < 0.05 and PB—5.1% (4.4–6.4%), *p* < 0.05, (Fig. 1).

**Fig. 2:**
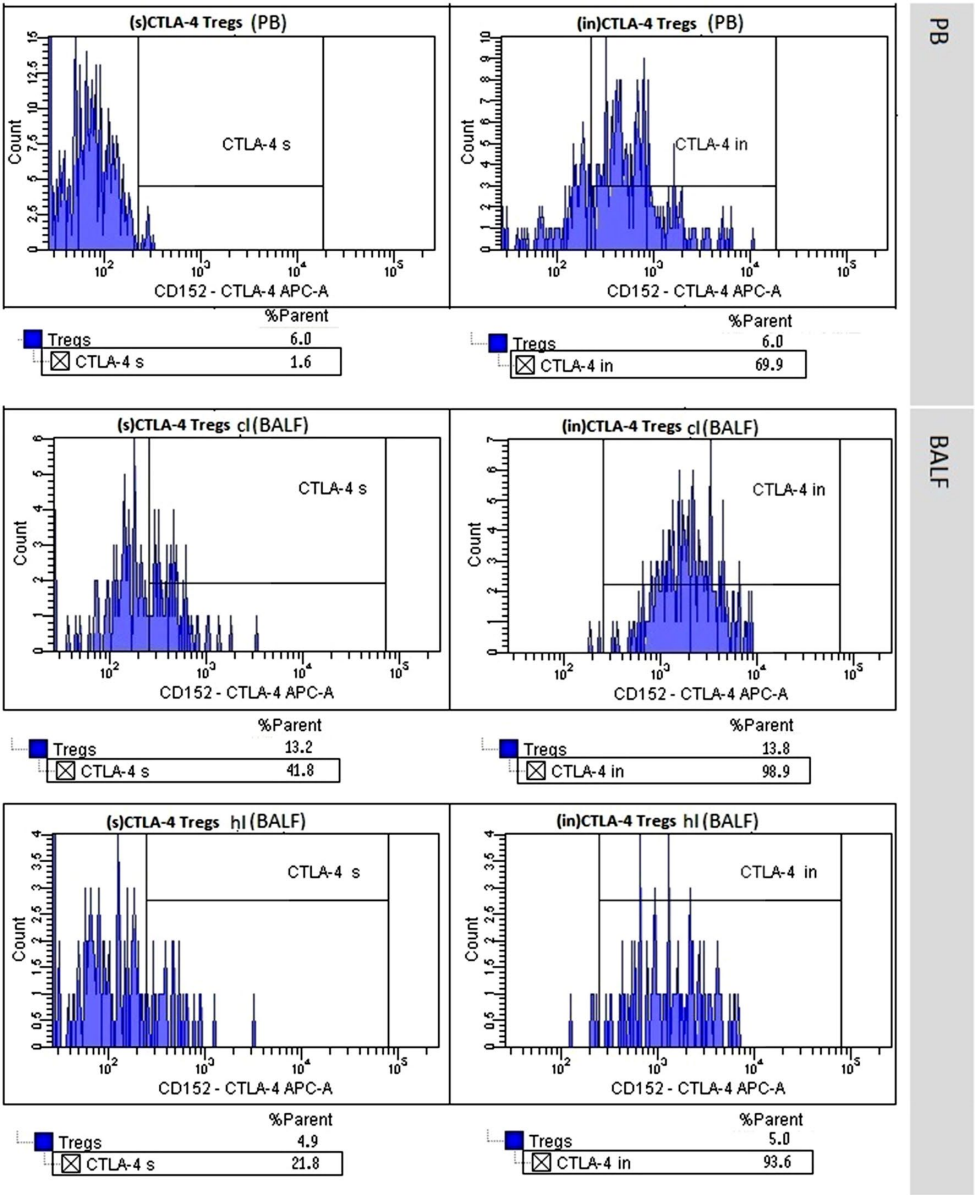
T regulatory cells (Tregs) with surface (s) and intracellular (in) presence of Cytotoxic T-lymphocyte antigen-4 (CTLA-4+ Tregs) in peripheral blood (PB), in bronchoalveolar lavage fluid from the lung affected by cancer (clBALF) and from healthy symmetrical lung (hlBALF) in one patient with lung cancer (example of histogram from flow cytometry analysis)

We noticed a higher proportion of (s)CTLA-4-positive Tregs in the clBALF when compared to the hlBALF and PB; it was 7.6% (3.8–13.3) versus 5.9% (3.3–7.1) versus 3.8% (2.0–8.9), respectively, difference nonsignificant. The median proportion of (in)CTLA-4-positive Tregs was also higher in the clBALF than in the hlBALF and PB 89.0% (83.2–96.3) versus 81.5% (61.7–92.0) versus 56.0% (54.1–60.7), respectively, *p* < 0.05, (Fig. 2, examples of flow cytometry gating of CTLA-4+ cells).

We calculated a ratio: (s)CTLA-4 to (in) CTLA-4 ((s) CTLA-4 Tregs/(in)CTLA-4 Tregs × 100%), and it was as follows: 8.1% (2.2–15.0) versus 6.9% (4.5–11.5) versus 6.5% (3.8–15.2) in the clBALF, hlBALF and PB, respectively, difference nonsignificant.

**Fig. 3:**
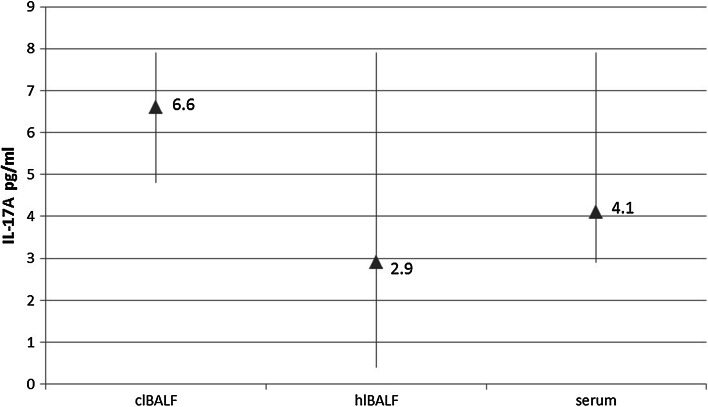
Differences between IL-17A concentration in bronchoalveolar lavage fluid supernatant from the lung affected by cancer (clBALF), from healthy symmetrical lung (hlBALF) and serum. Data are presented as median proportion and range p 25–75

The median proportion of IL-17A concentration in the clBALF supernatant was 6.6 pg/ml (5.4–8.8) and was higher than in the hlBALF supernatant: 2.9 pg/ml (0.4–7.9) and serum—4.1 pg/ml (2.9–7.9), difference nonsignificant (Fig. 3).

We observed a significant correlation between the proportion of Tregs and IL-17A concentration only in the clBALF (*r* = 0.5, *p* < 0.05). We did not observe this correlation in the hlBALF and serum.

**Fig. 4:**
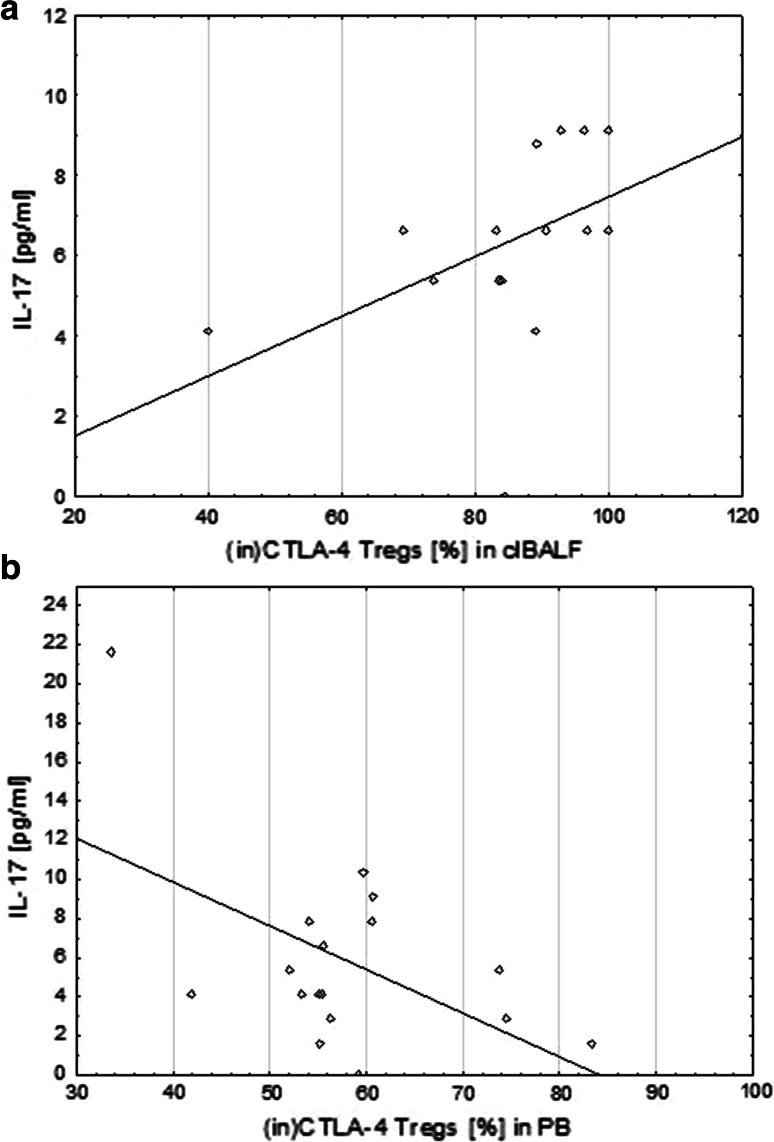
Correlation of the proportion of intracellular cytotoxic T-lymphocyte antigen-4 T regulatory cells ((in)CTLA-4+ Tregs) and concentration of IL-17A in bronchoalveolar lavage fluid from the lung affected by cancer (clBALF) (**a**) and opposite correlation in systemic environment in peripheral blood (PB)/serum (**b**)

There was also a positive significant correlation between the proportion of (in)CTLA-4+ Tregs and IL-17A concentration in the clBALF (*r* = 0.7, *p* < 0.05) but a significant negative correlation between the proportion of (in)CTLA-4+ Tregs and IL-17A concentration in the serum (*r* = −0.3, *p* < 0.05) (Fig. 4).

## Discussion

Lung cancer has a very insidious clinical course. Occurrence of resectable tumors does not exceed 30%; hence, the knowledge of the anticancer response and regulatory elements in the lung cancer environment is limited. It is also known that lungs show some individuality and specificity of the immune system and changes in the lung cancer environment are different than those observed in the systemic environment reflected by PB examination.

In our study, we focused on the assessment of selected regulatory elements of the immune response in lung cancer. To the best of our knowledge, it is the first study in which the panel of regulatory cells was identified in the BALF of lung cancer patients. We found significant differences of Tregs proportion and Tregs with (in) CTLA-4 expression between the local lung cancer environment and opposite lung and systemic response. We observed a significant correlation of the proportion of these cells with IL-17A concentration in the BALF from the cancer milieu. We confirmed the usefulness of BALF examination in this study. Our study fits in a broad trend of research of immunological markers as possible predictor factors in malignant tumors [30–32].

The study group consisted of patients with confirmed primary lung cancer in all stages of the disease and all types of NSCLC. The ratio of women to men was 1:2 which corresponds with literature data [33, 34]. 78% of patients were active smokers or ex-smokers. The mean number of pack-years among these patients was 36.3 ± 14.5. 78% of our patients did not report any symptoms of respiratory diseases. The remaining 22% of the study group confirmed the presence of symptoms such as cough, dyspnea and hemoptysis. Thus, the clinical characteristics of patients remains in accordance with the references and confirms the proper qualification of patients [1, 35]. It should be pointed that we followed the exclusion criteria to avoid any influence of extrinsic factors possibly modifying the immune system.

In this study, we observed the higher proportion of Tregs in the clBALF than in the hlBALF and PB. We identified the cells which are most closely to a functionally effective Tregs population defined by CD4+/CD25^high^/Foxp3+/CD127-. The CD4+/CD25^high^ cells have a strong suppressive activity [12], Foxp3 is connected *per se* with an inhibitory T cell function [13] and the lack of CD127 expression discriminates Tregs from effector T cells [14].

Similar results were noted by Erfani et al. [20]. In their study, a percentage of Tregs was significantly higher in the patients with NSCLC than in the healthy donors; however, this investigation concerned the systemic immune response. The percentage of Tregs in the PB of healthy subjects in the study of Erfani et al. was similar to the percentage of Tregs in the BALF from “healthy” lung and the PB in our study. In other studies, Liu et al. [36] and Okita et al. [37] presented a higher proportion of Tregs (CD4+ CD25+) in the PB of patients with NSCLC when compared with healthy subjects. However, Tregs were identified based on the presence of only 2 antigens: CD4+ CD25^high^ without Foxp3+. In this study, a higher percentage of Tregs in the PB of patients in an advanced stage of lung cancer than in the early stage of disease was shown [37]. In another study on patients with gastric cancer and esophageal cancer, a correlation between percentage of Tregs (CD4+ CD25^high^ with Foxp3 mRNA) with the severity of disease was observed in both cancers. The percentage of Tregs negatively correlated with patients’ survival [38]. A positive correlation of Tregs (CD4+ CD25^high^ and the Foxp3 mRNA) with the tumor stage was also noted in patients with hepatocellular carcinoma [39]. Similar relationships have been shown in other studies on tumor infiltrating lymphocytes (TIL), lymph nodes and PB lymphocytes. Shigematsu et al. [40] found a higher proportion of Tregs (CD4+ CD25+ Foxp3+) in TIL and lymph nodes than in PB and presented that Tregs suppressed the induction of cytotoxic T lymphocytes (CTLs) against lung cancer cells. Petersen et al. [41] showed that patients with stage I NSCLC who had a high proportion of Tregs in TIL were at a significantly higher risk of recurrence. The common conclusion from our results and other studies is that the proportion of Tregs in BALF, among TIL and in lymph nodes, is higher than in PB and indicates a role of these cells in the modulation of immune response in the lung cancer microenvironment.

In this study, we analyzed two forms of the CTLA-4 molecule on Tregs: surface and intracellular. We found a higher proportion of Tregs with the expression of CTLA-4 in the clBALF than in hlBALF and PB. The median proportion of (s)CTLA-4 Tregs was lower than (in)CTLA-4 Tregs in each analyzed material. CTLA-4 is rapidly endocytosed and accumulated in organelles [19]. The newly formed CTLA-4 molecules are stored in the structure of the Golgi apparatus and after activation are transported to the T cell surface, but next rapid endocytosis goes on [18]. Despite the relatively low surface expression of CTLA-4, it seems that a high concentration of intracellular CTLA-4 may deliver ready for rapid transport to the T cell surface and participate in the inhibition of lymphocyte activation. In our study, we observed a significantly higher proportion of (in)CTLA-4 Tregs in the clBALF than in the hlBALF and PB. The difference between the cancerous lung and peripheral blood was significant only for (in)CTLA-4 but not for (s)CTLA-4. This finding supports the role of intracellular domain in the process of immune response regulation. Erfani et al. [20] observed that an increased proportion of Tregs correlated with increased expression of the CTLA-4 molecule, which was related to the severity of the disease and poorer prognosis. In their study, the expression of CTLA-4 was analyzed on PB T cells, not Tregs. They showed a higher percentage of CD8+ cells with (s)CTLA-4 and a higher percentage of CD4+ cells with (in)CTLA-4 in the lung cancer patients as compared to the healthy subjects. Furthermore, this work, as well as our results, shows that the surface expression of CTLA-4 molecule is lower than (in)CTLA-4, independently of the type of T cell subpopulations. In another study, Kono et al. observed a higher percentage of Tregs (CD4+ CD25^high^) with intracellular expression of CTLA-4 in the PB of patients with stomach cancer and esophagus cancer and correlated with progression of the diseases [38]. Zheng et al. [42] showed a higher expression of CTLA-4 in NSCLC tissues than in normal tissue and no differences in relation to the histological type of cancer. To summarize it should be emphasized that our results on CTLA-4 on BALF Tregs cells are new and original observation without references in the literature.

In the present study, we also calculated the (s):(in)CTLA-4 ratio. This ratio can well characterize and reveal the movement of this molecule. This ratio was slightly higher in the BALF from the lung affected by cancer. This observation is important in the context of new anti-CTLA-4 treatment (ipilimumab) effective in lung cancer [43]. A high surface presentation of CTLA-4 may be an accessible target for such a therapy, especially for the action of anti-CTLA-4 antibodies in the local microenvironment. Thus, the confirmation of high expression of (s)CTLA-4 on the BALF cells may serve in the future as a possible biomarker. Our results confirmed a strong relationship between the CTLA-4 molecule and Tregs, both of them have an immunosuppressive function and the anti-CTLA-4 therapy is capable of targeting not only T effector cells but also regulatory T cells [10]. Further studies on CTLA-4 cellular traffic and the differences in the expression of surface versus intracellular domains in the cancer environment could contribute to finding an accurate biomarker for therapy.

In this study, we also showed the possible participation of IL-17 in regulation of antitumor immunity. We observed a higher median concentration of IL-17A in the lung cancer milieu when compared to opposite lung and PB. Moreover, we observed a positive significant correlation between the median proportion of Tregs and IL-17 A concentration only in the clBALF. There was a positive significant correlation between (in)CTLA-4+ Tregs and IL-17 concentration in the clBALF and a negative significant correlation between the median proportion of (in)CTLA-4+ Tregs and IL-17 A concentration in serum. Cantini et al. [44] in their experimental study showed the interconnection between Tregs and Th17 cells, which are the main source of IL-17. Their results suggested that the polarization of Th17 cells can be induced by Tregs and Th17 cells can modulate tumor development in the course of gliomas. Yang et al. [45] investigated Foxp3+ IL-17+ cells using flow cytometry in patients with colorectal cancer. They demonstrated a high expression of these cells in colon cancer tissue and suggested that the population of these cells may influence on cancer growth.

On the other hand, in our study we observed a negative significant correlation between the median proportion of (in)CTLA-4+ Tregs and IL-17 A concentration in the systemic environment. Some studies indicated possible anti-tumor activity of IL-17 by the stimulation of specific effective immune response against the tumor cells and tumor growth inhibition. Kryczek et al. [21] showed that IL-17 may be involved in the recruitment of effector CD8+ T cells by stimulation of the production of CXCL9 and CXCL10 in patients with ovarian cancer. They presented a negative correlation of tumor infiltrating Tregs and the level of IL-17 with the severity of the disease. Vasilescu et al. [46] analyzed the expression of Foxp3+ and IL-17 by immunohistochemistry as a potential target for cancer immunotherapy in resected lung adenocarcinomas. Foxp3 and IL-17 were present in TIL, tumor cells and fibroblasts; IL-17 was expressed also in periendothelial cells. The reaction in tumor cells was weaker than in other cells. A negative correlation between lymphocytes Foxp3+ and IL-17+ periendothelial cells was observed, suggesting some antagonism. Similar results were obtained by Zhang et al. [47] who analyzed the ratio of Th17/Tregs in PB by flow cytometry and the serum IL-17 concentration by ELISA in patients with NSCLC. They showed that the Th17/Tregs ratio decreased in the patients with lung cancer compared to the healthy subjects and showed a negative correlation with the clinical stage of the disease. In addition, serum IL-17 concentration was lower in the patients with NSCLC than in the control group. Our results and the results of other authors indicate that Th17 cells and Tregs may have synergistic function in the tumor microenvironment promoting cancer growth while the systemic role of IL-17 requires understanding of the dual function of this interleukin.

## Conclusion

The most striking finding of our study was the confirmation of differences in the presence of regulatory cells between three compartments: the cancerous lung, healthy lung and PB reflected, respectively, by: the clBALF, hlBALF and PB derived from the same patient. The proportion of CD4+/CD25^high^/Foxp3+/CD127- Tregs determined by flow cytometry and the proportion of Tregs with expression of (in)CTLA-4 were significantly elevated in the BALF harvested from the lung affected by lung cancer when compared with the last two compartments. Moreover, we observed a significant correlation between the proportion of Tregs and (in)CTLA-4+ Tregs and IL-17A concentration only in clBALF. Our findings emphasize the usefulness of BALF analysis in the assessment of immune status of a lung cancer patient before therapy, which may serve for determination of possible biomarkers.

## Abbreviations

(in)CTLA-4: Intracellular-expressed CTLA-4
(s)CTLA-4: Surface-expressed CTLA-4
BALF: Bronchoalveolar lavage fluid
clBALF: Cancer affected lung BALF
COPD: Chronic obstructive pulmonary disease
CTLA-4: Cytotoxic T-lymphocyte antigen 4
CTLs: Cytotoxic T lymphocytes
ELISA: Enzyme-linked immunosorbent assay
Foxp3: Forkhead box P3 transcription factor
hlBALF: Healthy symmetrical lung BALF
IL: Interleukin
MGG: May-Grunwald Giemsa
NOS: Not otherwise specified
NSCLC: Non-small cell lung cancer
PB: Peripheral blood
Th17: T helper 17
TIL: Tumor infiltrating lymphocytes
Tregs: Regulatory T cells
